# The physiological and molecular response of *Aurelia* sp.1 under hypoxia

**DOI:** 10.1038/s41598-017-01318-x

**Published:** 2017-05-08

**Authors:** Guoshan Wang, Yu Zhen, Zhigang Yu, Yan Shi, Qing Zhao, Jianyan Wang, Tiezhu Mi

**Affiliations:** 1 College of Environmental Science and Engineering, Ocean University of China, Qingdao, Shandong P.R. China; 2 National Marine Hazard Mitigation Service, Beijing, China; 3 Key Laboratory of Marine Environment and Ecology, Ministry of Education, Qingdao, P.R. China; 4 Laboratory for Marine Ecology and Environmental Science, Qingdao National Laboratory for Marine Science and Technology, Qingdao, P.R. China; 5 Key Laboratory of Marine Chemistry Theory and Engineering, Ministry of Education, Qingdao, P.R. China; 6 Beijing Museum of Natural History, Beijing, P.R. China; 7 Zhongtian Technology Marine Systems Co., Ltd., Nantong, P.R. China

## Abstract

Few studies have been published on the mechanisms of hypoxia response and tolerance in jellyfish, especially with respect to the regulatory mechanism at the molecular level. In this study, *Aurelia* sp.1, which is frequently found in Chinese coastal waters, was cultivated in a hypoxic system to determine the molecular mechanisms underlying its hypoxic response by studying the physiological activity, gene expression and metabolite contents in the prolyl hydroxylase domain (PHD)-hypoxia inducible factor (HIF) oxygen-sensing system. Physiological activity; the expression of *PHD*, *HIF*, *ALDO* (fructose-bisphosphate aldolase), *PDK* (pyruvate dehydrogenase kinase), and *LDH* (lactate dehydrogenase) genes; and the lactic acid content in medusae were significantly affected by hypoxia. The up-regulation of *ALDO*, *PDK* and *LDH*, which was directly or indirectly induced by HIF, mediated the transition from aerobic respiration to anaerobic glycolysis in the medusae. In polyps, there was a slight increase in the expression of *HIF*, *PHD* and *ALDO*, no obvious change in that of *PDK* and a slight decrease in that of *LDH* throughout the experiment; however, these changes were insufficient to induce the shift. This study provides a scientific basis for elucidating the regulatory mechanism underlying the PHD-HIF oxygen-sensing system in *Aurelia* sp.1.

## Introduction

Seasonal hypoxia events occur frequently in summer in estuaries, bays, and coastal waters, such as those in the northern Gulf of Mexico, Baltic Sea, Bering Sea, Hiroshima Bay, Western Seto Inland Sea, Tokyo Bay, and the Yangtze Estuary and adjacent areas. Coincidentally, jellyfish populations have bloomed almost every year in these waters. In particular, areas that experience prolonged hypoxia, such as the northern Gulf of Mexico, have large jellyfish populations^1^, and moon jellyfish gather in the coastal areas of northern Hiroshima Bay where bottom hypoxia occurs^2^. Thus, there appears to be an association between marine hypoxia events and jellyfish blooms. As Cnidarians, jellyfish can survive and reproduce under hypoxic conditions better than other marine animals. Therefore, many reports mention that the abilities associated with hypoxic tolerance may provide jellyfish with competitive advantages in marine ecosystems.

The scyphozoan genus *Aurelia*, commonly called moon jellyfish, is widely distributed throughout coastal waters worldwide. Moon jellyfish are the dominant jellyfish species in blooms occurring in Chinese coastal waters (*Aurelia* sp.1, according to molecular identification methods based on 18S and ITS-5.8S rDNA sequences)^3^. The life cycle of *Aurelia* generally consists of the asexual generation of polyps and the sexual generation of medusae. Sessile polyps tightly attach to suitable hard substrates in certain waters, whereas pelagic medusae can migrate vertically and horizontally in response to tides and currents. Studies have revealed that both polyps and medusa exhibit hypoxic tolerance. The bell contraction rate of moon jellyfish medusae does not vary with the oxygen concentration in the tested range (1.0, 2.0, 4.0, and 5.8 mg/L), but the predation rates at oxygen concentration of 1.0 and 2.0 mg/L are significantly higher than those at 4.0 and 5.8 mg/L^4–6^. The median survival time of moon jellyfish polyps at an oxygen concentration of 0.28 mg/L is 120 hours, and moon jellyfish polyps can normally survive and reproduce at 2.8 mg/L^7^.

Although jellyfish are known to thrive under hypoxia, the physiological and molecular mechanisms involved in their hypoxic tolerance remain unknown. Rutherford and Thuesen demonstrated that jellyfish can use mesoglea as oxygen reservoirs under hypoxia. However, intragel oxygen present in the mesoglea is exhausted by medusae after approximately 2 hours of anoxia^8, 9^. Thus, this physiological mechanism confers only short-term protection against hypoxia for medusa, unlike polyps. Thus, other mechanisms involve the ability of tissues and cells to enhance their utilization of oxygen under very low ambient partial pressures. Three modes of adaptation to hypoxia existing in marine organisms have been proposed to compensate for the difference between aerobic respiration capacity and total metabolic demand: the development of mechanisms for the highly effective utilization of oxygen from water, the metabolic rate reduction and the use of anaerobic glycolysis^10^. However, jellyfish, which are considered the earliest eumetazoans, have not yet evolved respiratory and vascular systems. It is not possible for jellyfish to adapt to hypoxia by increasing their gill ventilation rate as Spanish mackerel (*Scomberomorus niphonius*) do or to increase the oxygen affinity of haemocyanin as Crustaceans do^4^. Therefore, metabolic regulation may be crucial for the hypoxic tolerance of jellyfish.

In mammals and fish, metabolic regulation is a principal and primordial function of hypoxia-inducible factors (HIFs) under hypoxic conditions^11^. HIF, a heterodimeric transcription factor, was first identified on the 3′ enhancer of the erythropoietin gene in Hep3B cells and can regulate many fundamental processes, including glycolysis, the tricarboxylic acid (TCA) cycle and special oxygen delivery systems in the cells of higher animals^12, 13^. The expression of this factor is induced by declining oxygen concentrations. HIF exists in all extant metazoan species analysed to date^14^. Not surprisingly, Wang *et al*. identified an analogue of HIF in *Aurelia* sp.1 and showed that the expression of HIF increased not only at the translational level but also at the transcriptional level under hypoxia^15, 16^. However, under normoxic conditions, HIF is hydroxylated by prolyl hydroxylase domains (PHDs) and is then subjected to ubiquitination and proteasomal degradation via the von Hippel-Lindau protein. As members of an evolutionarily conserved subfamily of dioxygenases, PHDs catalyse the hydroxylation of HIF using oxygen and 2-oxoglutarate as co-substrates and iron and ascorbate as cofactors and thus can serve as direct sensors of cellular oxygen tension. More interestingly, HIF can bind to the hypoxic response elements of *PHD* genes and up-regulate their transcriptional expression under hypoxia^17^. In all animal taxa examined to date, PHD and HIF constitute the PHD-HIF oxygen-sensing systems that monitor and respond to fluctuations of cellular oxygen to maintain physiological oxygen homeostasis^18^. It follows that this central physiological regulatory system links dynamic variations in cellular oxygen concentration and the regulation of related fundamental processes, especially in altering materials and energy metabolism to improve the prospects of survival under hypoxia. This regulation is achieved by up-regulating glycolytic and anaerobic glycolysis while inhibiting oxidative metabolism, which is characterized by changes in the gene expression of three key factors: fructose-bisphosphate aldolase (ALDO), pyruvate dehydrogenase kinase (PDK), and lactate dehydrogenase (LDH)^19–22^. ALDO (EC 4.1.2.13) is an important glycolytic enzyme that catalyses the cleavage of the aldol- fructose 1,6-bisphosphate into triose phosphate dihydroxyacetone phosphate (DHAP) and glyceraldehyde 3-phosphate (G3P). PDK (EC 2.7.11.2) phosphorylates and inactivates pyruvate dehydrogenase (PDH), thereby inhibiting the conversion of pyruvate to acetyl coenzyme A (acetyl-CoA) for entry into the tricarboxylic acid cycle, which generates energy through the oxidation of acetyl-CoA. LDH (EC 1.1.1.27) catalyses the reversible conversion of pyruvate and lactate.

Studies of the metabolic processes mentioned above are critical for improving our understanding of the physiological and molecular responses of jellyfish under hypoxic conditions and for further clarifying the links between marine hypoxia and jellyfish blooms. Accordingly, we cultivated *Aurelia* sp.1 in a hypoxic system and measured physiological activity and related gene expression and metabolite (lactic acid) content based on PHD-HIF oxygen-sensing systems and key metabolic nodes.

## Results

### Dissolved oxygen concentration profiles

**Figure 1:**
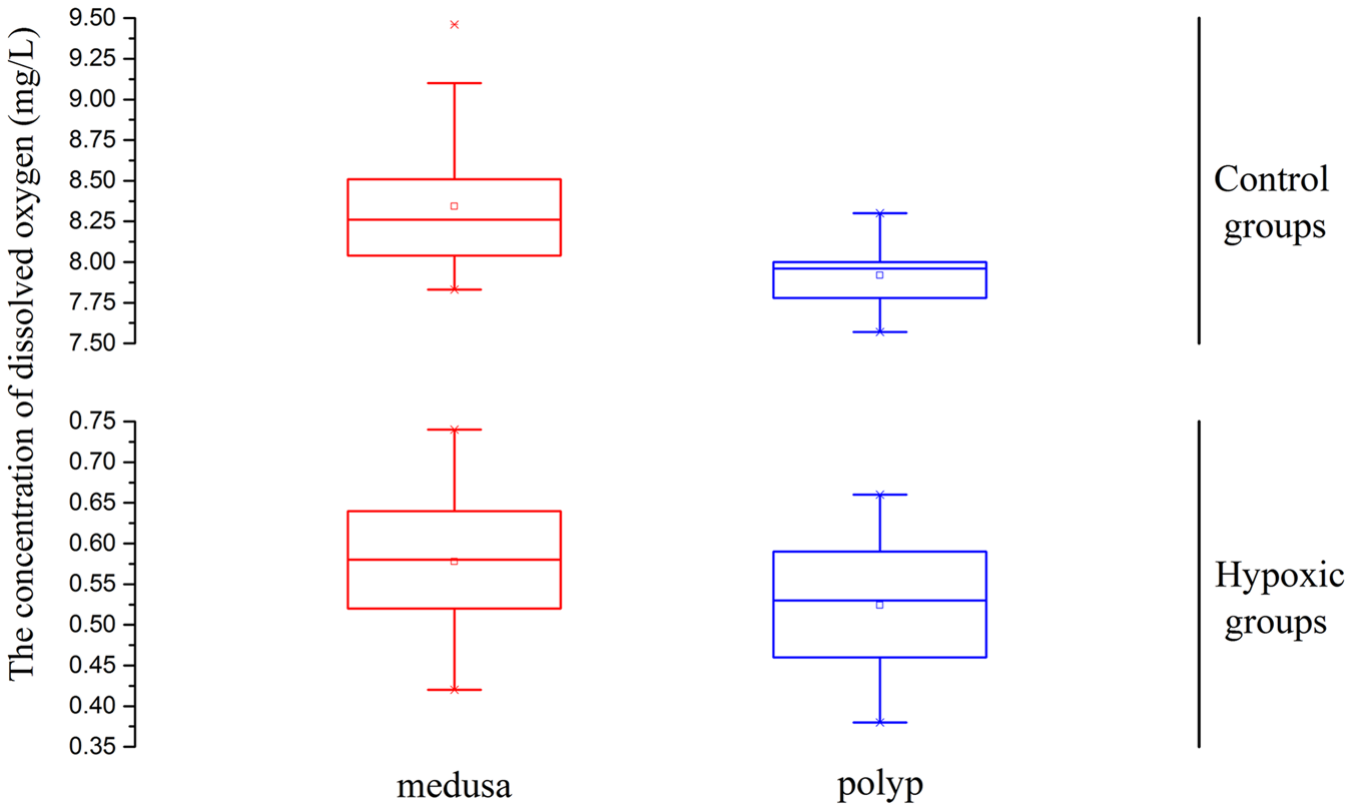
The dissolved oxygen concentration range.

The medusa-hypoxic and polyp-hypoxic groups were maintained at dissolved oxygen (DO) concentrations of 0.58 ± 0.07 (mean ± SD) and 0.52 ± 0.07 mg/L throughout the experiment, respectively, whereas the DO concentration for the control groups was higher than 7.50 mg/L (Fig. 1). The hypoxic jellyfish cultivation system was able to provide stable low-oxygen conditions for studying the molecular mechanisms underlying hypoxia response and tolerance.

### Variation in the number of bell contractions

**Figure 2:**
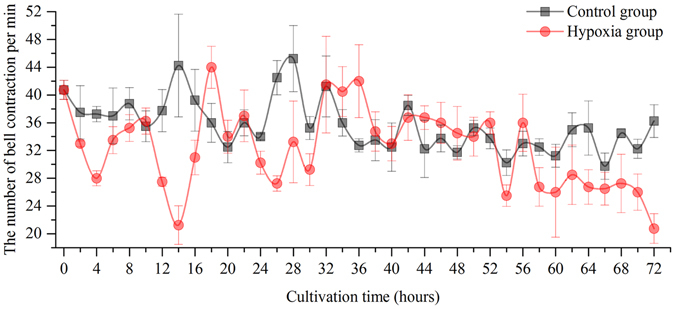
The variation of bell contraction number per minute in medusa.

The mean number of bell contractions in the control group was 35 ± 0.50 (mean ± SE) per min and did not vary as the cultivation time was extended during the experiment; however, the mean number of bell contractions in the hypoxic group was 32 ± 0.66 per min. There was a significant effect of the hypoxic treatment on the bell contraction rate of jellyfish (Wilcoxon test, *p*-value < 0.01). As shown in Fig. 2, the bell contraction number in the hypoxic group decreased after the experiment started; this number decreased to 21 per min at 14 hours, sharply increased to 44 per min at 18 hours, decreased again until 30 hours, significantly increased to 40 per min at 32 hours (four hours), and later decreased to 35 per min for twenty hours. After 58 hours, the bell contraction number decreased until the end of the experiment. These results strongly suggest that the physiological activity of the medusa was significantly affected by the oxygen levels of the sea water, and different variation trends were seen during cultivation.

### The gene expression of the PHD-HIF oxygen sensing system under hypoxia

**Figure 3:**
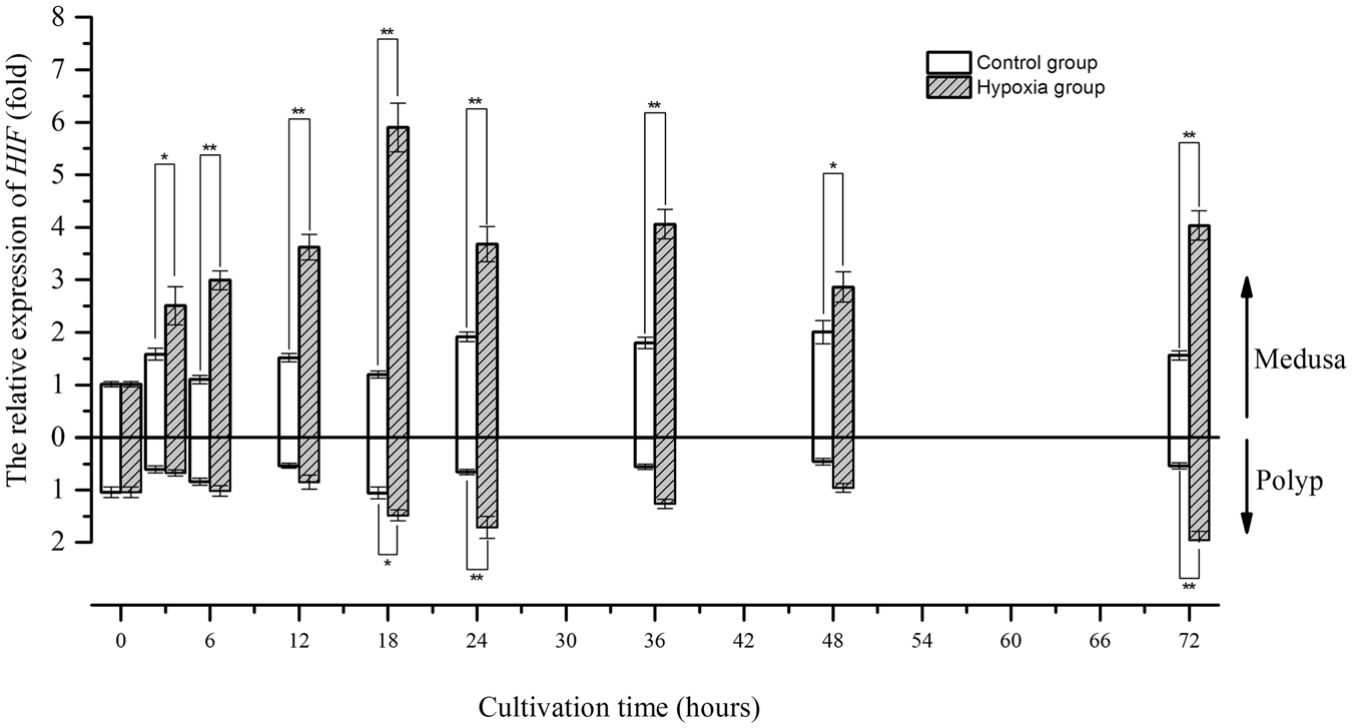
The gene expression variation of *HIF* in medusa and polyps over time (*represents *p* < 0.05 and **represents *p* < 0.01; Error bar is standard error).

**Figure 4:**
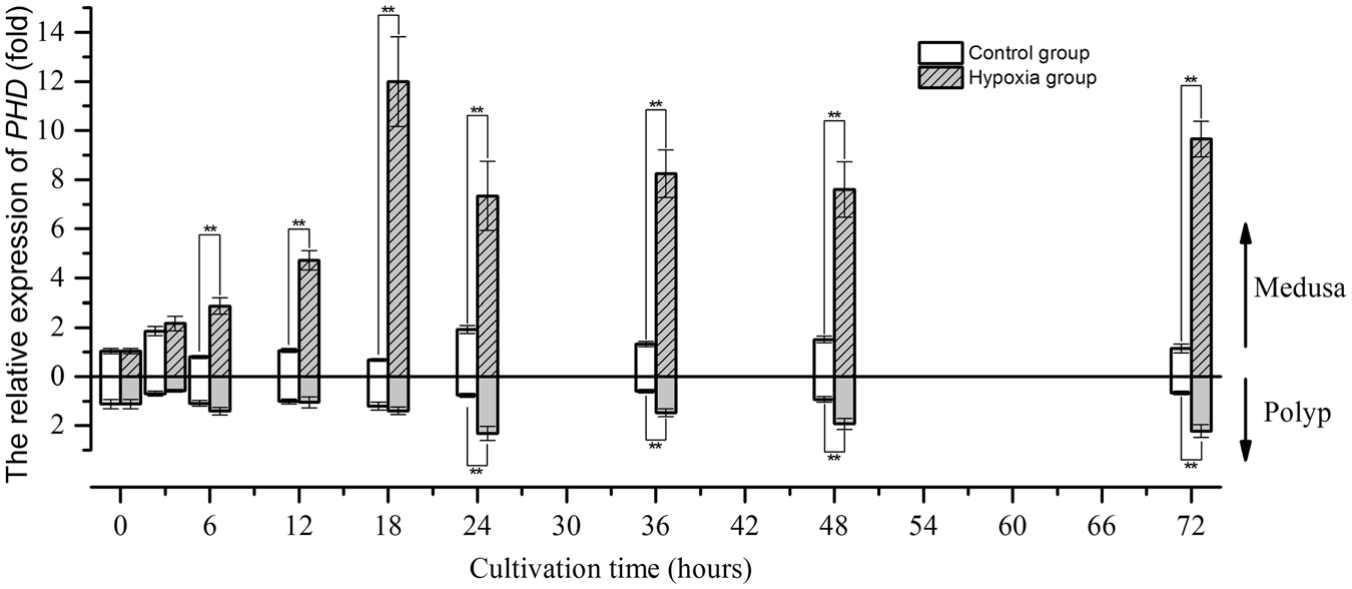
The gene expression variation of *PHD* in medusa and polyps over time (*represents *p* < 0.05 and **represents *p* < 0.01; Error bar is standard error).

The relative expression of *HIF* increased approximately 1.5-fold (mean) at 3 hours compared with the value at 0 hours and remained stable until the end of the experiment in the control group of medusae. In contrast, the relative expression of *HIF* in the control group of polyps decreased approximately 0.6-fold (mean) compared with the value at the beginning of the experiment and remained stable until the end of the experiment (Fig. 3). In low-oxygen groups, the relative expression of *HIF* in the medusae significantly increased after 3 hours, peaked at 18 hours (approximately 4.92-fold compared with that in the control group, Welch two -sample t-test, *p*-value < 0.01), and subsequently remained at a high level. The relative expression of *HIF* in the polyps continued to significantly increase after 18 hours to a value 1.7-fold greater than the initial value at 24 hours (approximately 2.6-fold higher than that in the control group, Welch two-sample t-test, *p*-value < 0.01) and decreased gradually thereafter until 48 hours; the highest level of *HIF* expression appeared at 72 hours (approximately 2.6-fold higher than that in the control group, Welch two-sample t-test, *p*-value < 0.01) and was 1.95-fold higher than the initial value. The variation in *PHD* expression over time was similar to that of *HIF*; however, the relative expression of *PHD* increased after 6 hours in medusae but after 24 hours in polyps (Fig. 4). Based on the above results, it is clear that both *HIF* and *PHD* respond to environmental hypoxia at the transcriptional level in jellyfish. Furthermore, the sensitivity of the *PHD* response to hypoxia was lower than that of *HIF* due to the time delay in the up-regulation of gene expression. Moreover, the observed changes in gene expression also showed that the hypoxic sensitivity of the polyps was weaker than that of medusae at the same DO level.

### Metabolism-related gene expression under hypoxia

**Figure 5:**
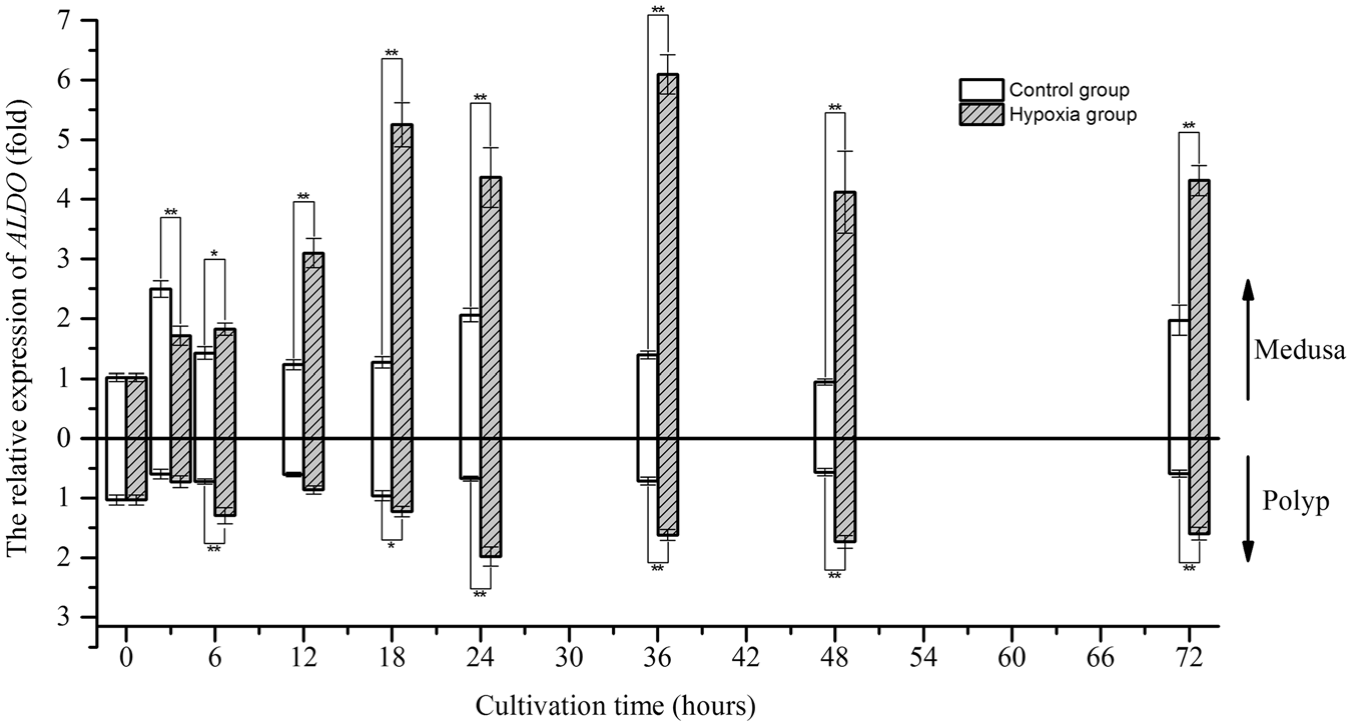
The gene expression variation of *ALDO* in medusa and polyps over time (*represents *p* < 0.05 and **represents *p* < 0.01; Error bar is standard error).

**Figure 6:**
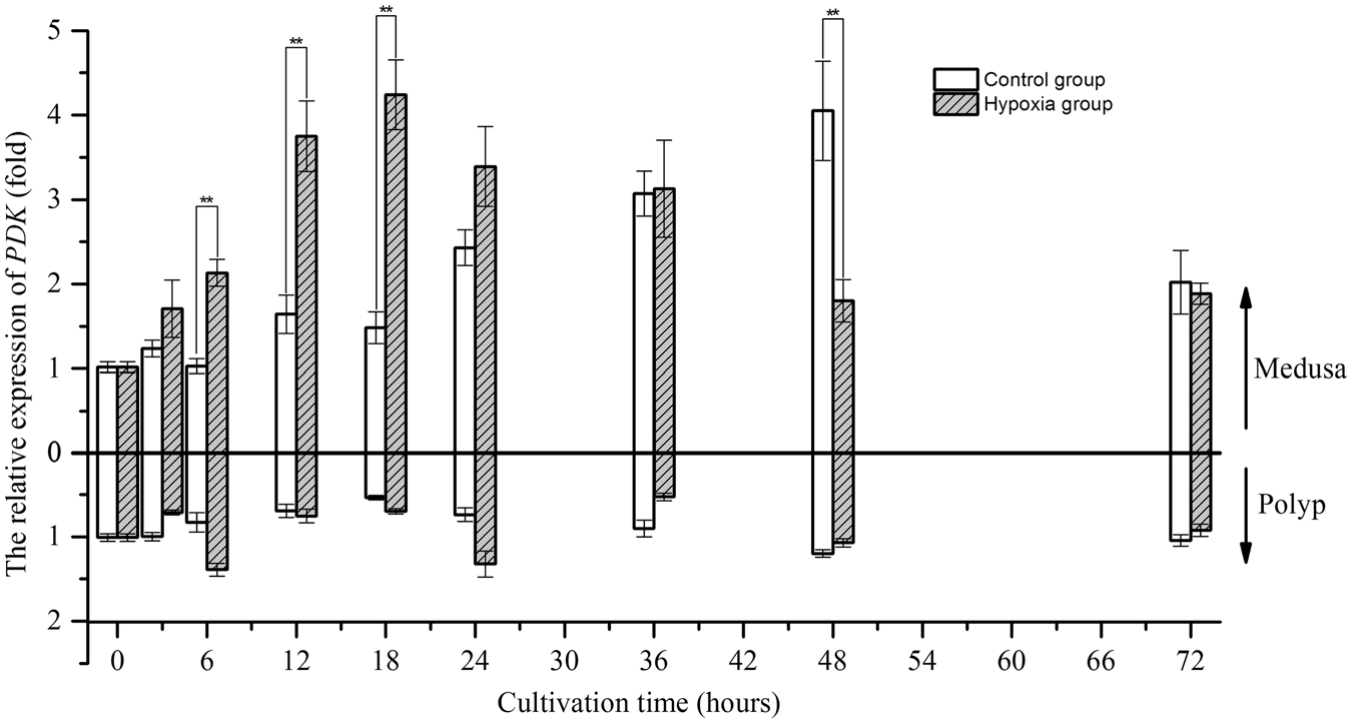
The gene expression variation of PDK in medusa and polyps over time (*represents p < 0.05 and **represents p < 0.01; Error bar is standard error).

**Figure 7:**
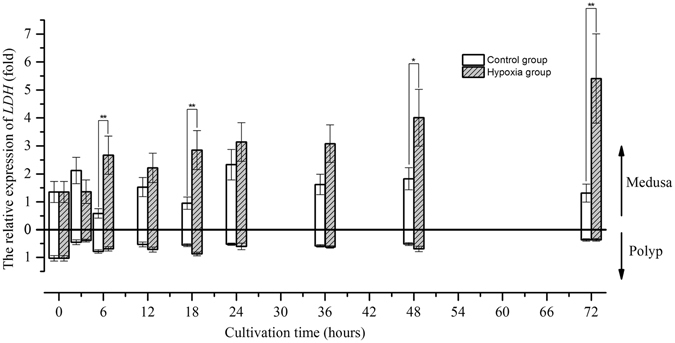
The gene expression variation of *LDH* in medusa and polyps over time (*represents *p* < 0.05 and **represents *p* < 0.01; Error bar is standard error).

After 3 hours, the relative expression of *ALDO* increased to a value 1.5-fold greater than that at 0 hours and fluctuated around that level in the control group of medusae, whereas the relative expression of *ALDO* in polyps decreased to a value 0.6-fold lower than that at the beginning of the experiment and then remained stable until the end of the experiment (Fig. 5). Under hypoxia, *ALDO* expression increased after 6 hours and then remained high in both medusa and polyps; these values were significantly different from those in the control group. However, the trend in the relative expression of *PDK* fluctuated once within a narrow range in the polyps. Therefore, we did not consider hypoxia to have induced significant changes in *PDK* expression relative to the control in polyps (Wilcoxon test, *p*-value > 0.05) (Fig. 6). In medusae, the changes in *PDK* expression were complex as cultivation time progressed. *PDK* expression in the control group increased after 24 hours, peaked at 48 hours, and decreased at 72 hours. In contrast, *PDK* expression in the hypoxic group increased after 6 hours, peaked at 18 hours, and decreased gradually thereafter. There were significant differences between the hypoxic and control groups at 6, 12, 18, and 48 hours. For polyps, the relative expression of *LDH* in both the control and hypoxic groups decreased to a value 0.5-fold lower than the starting level after 3 hours and fluctuated around this value until the end of the experiment (Fig. 7). In contrast, in medusae, *LDH* expression increased in the hypoxic group after 6 hours, unlike that in the control group, which fluctuated around the initial value throughout the experiment. In summary, the expression levels of the three metabolism-related genes confirmed that the medusae were more sensitive to hypoxia than were the polyps, likely contributing to the differences in the response mechanisms.

### Change in lactic acid content

**Figure 8:**
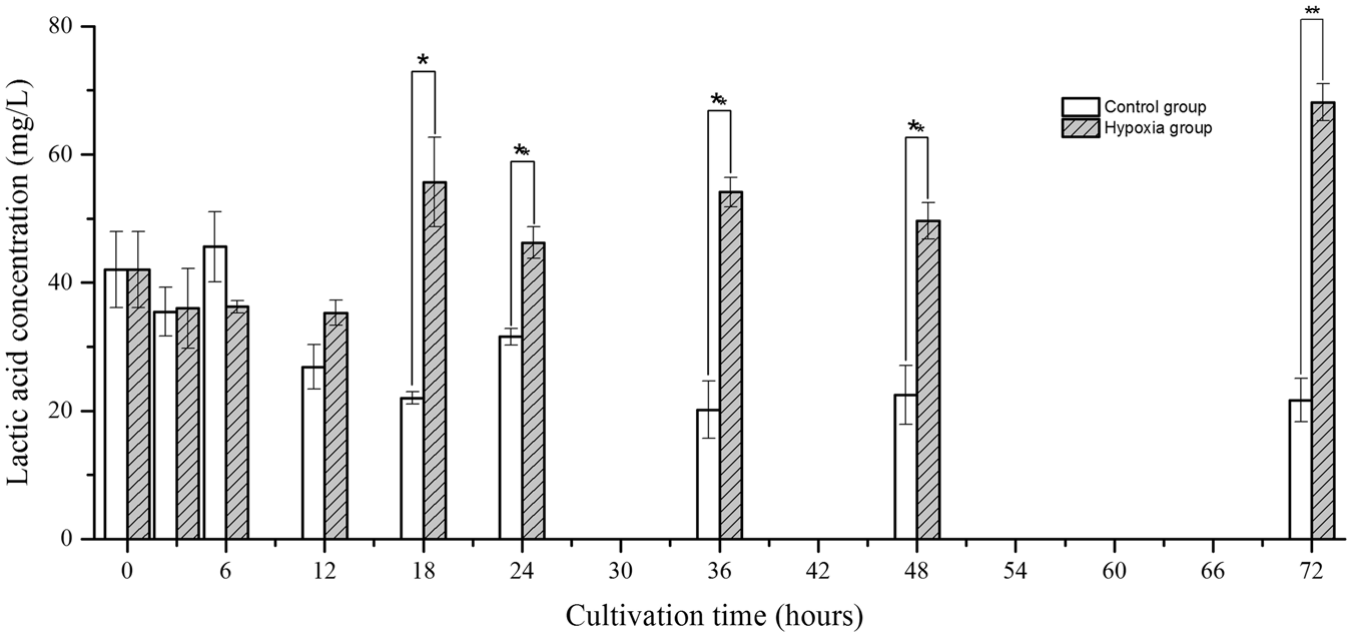
The variation of lactic acid content in medusa over time (*represents *p* < 0.05 and **represents *p* < 0.01; Error bar is standard error).

As shown in Fig. 8, the lactic acid content of the medusae in the control group gradually decreased from 0 to 12 hours and then reached a stable level of approximately 21.6 mg/L after 18 hours, whereas that of the medusae in the hypoxic group significantly increased after 18 hours and peaked at 72 hours. Thus, environmental hypoxia clearly induced an increase in the lactic acid content of the medusae.

### Correlation analysis of the five genes

**Figure 9:**
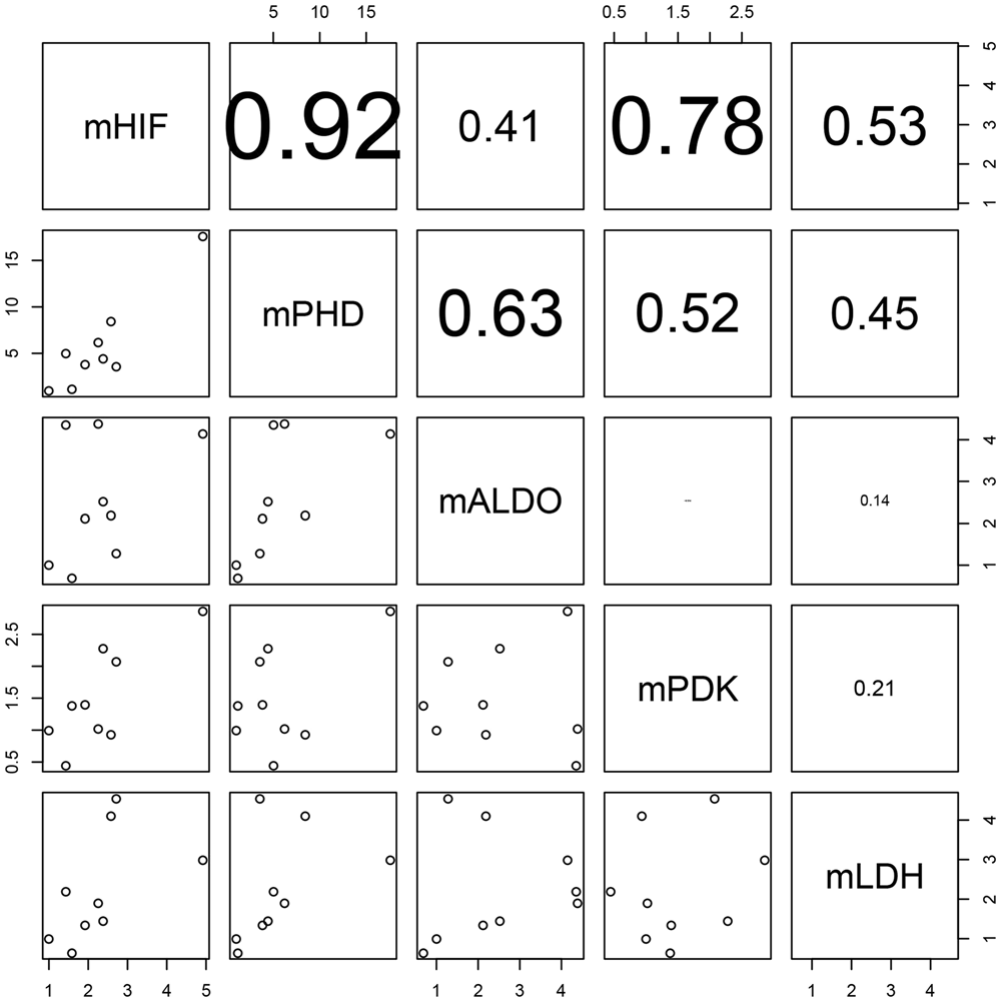
Correlation analysis of related gene expression in medusa.

**Figure 10:**
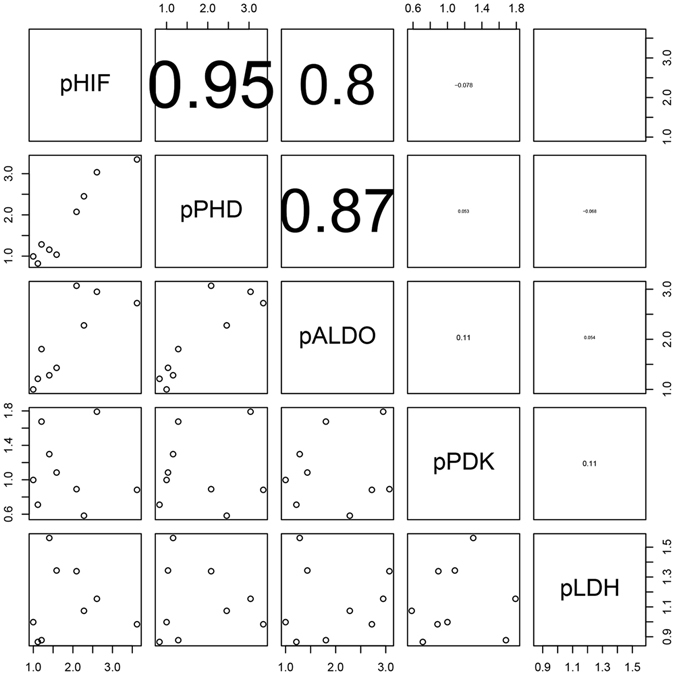
Correlation analysis of related gene expression in polyps.

Correlation analysis suggested that *HIF* gene expression in the medusae was strongly and positively correlated with *PHD* gene expression (*r* = 0.92) and exhibited a moderate and positive correlation with *PDK* and *LDH* expression; a weak but positive correlation was found between *HIF* and *ALDO* expression (0.41). Similarly, *PHD* gene expression exhibited specific relationships with the expression levels of *ALDO*, *PDK*, and *LDH* (Fig. 9). In the polyps, *HIF* gene expression was a significantly and positively correlated with *PHD* gene expression (0.95) and *ALDO* gene expression (0.80). *PHD* gene expression was strongly and positively correlated with *ALDO* gene expression (0.87) (Fig. 10); however, there were no positive correlations between *ALDO*, *PDK*, and *LDH* in either the medusae or polyps. These results indicate a direct association between the *HIF* and *PHD* genes in jellyfish under hypoxia; however, although *ALDO*, *PDK*, and *LDH* genes were affected by the PHD-HIF oxygen-sensing system, they remained independent of each other.

## Discussion

**Figure 11:**
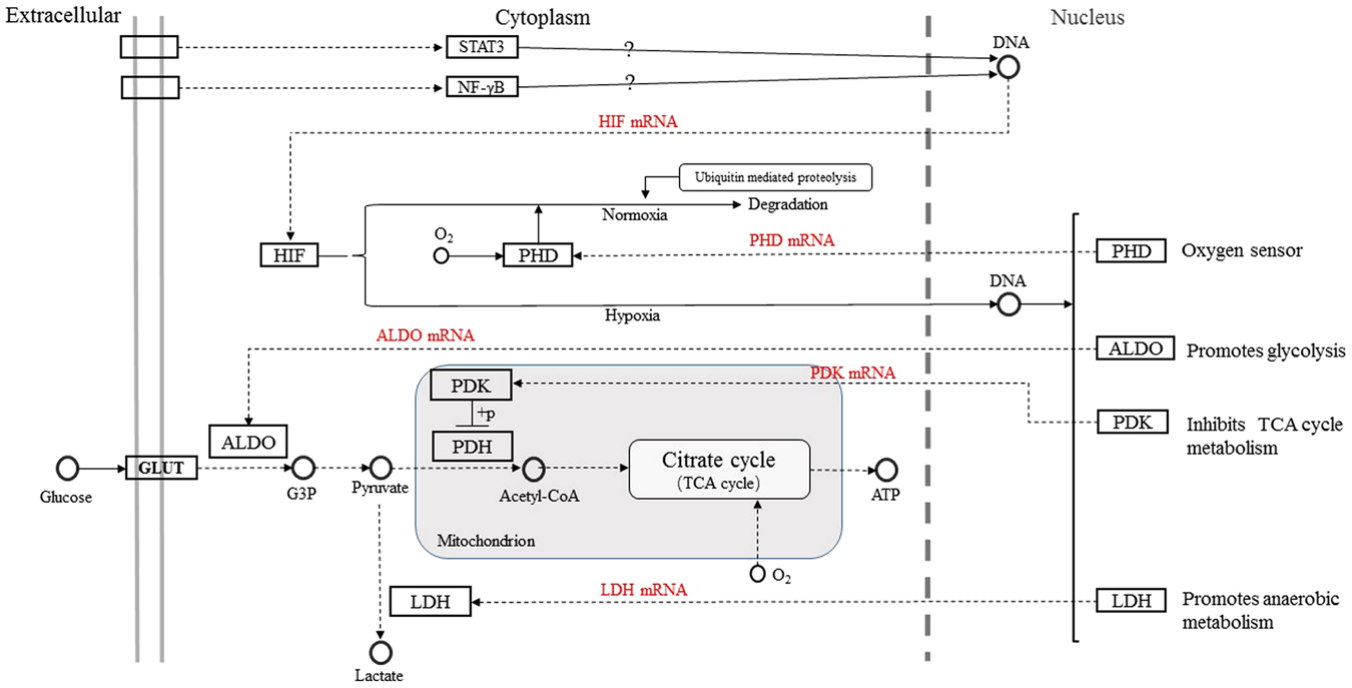
The partial regulation pathway of HIF (STAT3:signal transducer activator of transcription 3; NF-κB: nuclear factor κB; PHD: prolyl hydroxylase domains; HIF: hypoxia inducible factor; ALDO: fructose-biphosphate aldolase; PDK: pyruvate dehydrogenase kinase; LDH: lactate dehydrogenase; PDH: pyruvate dehydrogenase; G3P: glyceraldehyde 3-phosphate; GLUT: glucose transporter).

In mammalian cells, the PHD-HIF oxygen-sensing system is a sophisticated regulatory mechanism that responds to oxygen fluctuations and maintains cellular oxygen homeostasis (Fig. 11). Wenger *et al*. confirmed that HIF-1α is regulated at the post-mRNA level^23^. Subsequently, it was found that PHD and the asparaginyl hydroxylase factor inhibiting HIF (FIH) catalyse post-translational hydroxylation under normoxia, which leads to the proteasomal degradation of HIF via the von Hippel–Lindau protein or the blocking of its association with the p300 coactivator protein to reduce its transcriptional activity in turn^11^. In other words, the transcriptional activity of HIF depends on its own stability and accumulation within cells under hypoxia rather than its up-regulation at the mRNA level; however, another study revealed that HIF induces the expression of M2 isoform of pyruvate kinase (PKM2) and signal transducer activator of transcription 3 (STAT3), which in turn induce the transcriptional expression of HIF in highly proliferating cancer cells^24^. Uden *et al*. also demonstrated that tumour necrosis factor α (TNFα)-induced nuclear factor κB (NF-κB) can increase the expression of HIF-1α mRNA and protein and increases its activity level, indicating that cross-talk may occur between the NF-κB and HIF signalling pathways^25^. Jiang *et al*. observed an increase in HIF-1α mRNA levels in the lung, heart, kidney, and muscle tissues of Taiwan voles (*Microtus kikuchii*) under hypoxic stress with cobalt chloride^26^. In invertebrates, HIF homologues exhibit transcriptional responses to prolonged hypoxia in the gills of *Crassostrea virginica* ^27^. This experiment not only confirmed the transcriptional response of *HIF* mRNA to hypoxia at the medusa stage of *Aurelia* sp.1, as discovered by Wang *et al*.^16^, but also revealed the same result at the polyp stage^16^. Although the transcriptional response of HIF to hypoxia has been demonstrated in many organisms, the mechanism by which the expression of HIF mRNA is up-regulated in response to low oxygen concentration remains unknown. Therefore, it is necessary to compare the transcriptome of jellyfish under normoxia and hypoxia to illustrate the above mechanism. Our study indicated that the transcriptional expression of the *PHD* gene was strongly and positively correlated with that of the *HIF* gene, consistent with the previous findings of Cioffi *et al*. in human cardiovascular cells^28^. These findings further confirmed that HIF can prompt the transcriptional expression of the *PHD* gene (termed the HIF/PHD feedback loop) to limit the accumulation of HIF that is caused by hypoxia^29^. Thus, it was concluded that HIF accumulated due to the combined action at the transcriptional and post-translational levels. Under hypoxic conditions, HIF mediates a transition from aerobic respiration to anaerobic glycolysis by up-regulating the expression of three key factors, ALDO, PDK, and LDH, in higher animals; however, our results showed that the regulatory mechanism of HIF in jellyfish differs somewhat from that in higher animals under hypoxia. A definite positive correlation was found between the transcriptional expression of *HIF* and *ALDO* in both medusae and polyps. Considering the trends found in their expression levels over time, it is believed that the transcriptional expression of the *ALDO* gene is likely regulated by the transcription factor HIF in jellyfish. The increase in ALDO expression not only accelerates glycolysis to generate adenosine triphosphate (ATP) but also prevents the excess mitochondrial generation of reactive oxygen species (ROS) in cells under hypoxia^30, 31^. PDK plays a pivotal role in the control of metabolic flexibility under various nutrient conditions in human cells^32^. The expression of *PDK* is regulated not only by HIF but also by several nuclear hormone receptors, including peroxisome proliferator-activated receptors (PPARs), glucocorticoid receptors (GRs), and thyroid hormone receptors (THRs)^33–35^. Regarding the *PDK* gene studied here, gene expression was correlated with *HIF* expression in medusae but not in polyps at an oxygen concentration of 0.5 mg/L, and the trend in expression was similar to that of *HIF* in the hypoxia group. These results suggest the complexity of *PDK* gene regulation; this regulation was mainly affected by HIF under hypoxia but was also affected by other factors under normoxia. *LDH* gene expression was increased under hypoxic conditions over time, similar to the changes in lactic acid content, and the gene expressions of *LDH* and *HIF* were correlated in medusae but not in polyps. These results imply that *LDH* gene expression in jellyfish may be indirectly regulated by HIF under hypoxia and that the observed increase in *LDH* mRNA may be driven by pyruvic acid accumulation.

Obvious differences exist between polyps and medusae, such as their shape, anatomical organization, and set of somatic cells. The umbrella of medusae have a layer of mononucleated striated muscle cells that can support their movement, whereas the polyps only attach to stationary substrates and depend on telescopic nematocysts to capture prey. The energy required for movement is obtained from ATP, which is generated by the oxidation of glucose and fatty acids in the mitochondria. Therefore, it can be inferred that the physiological and molecular response of pelagic medusae is stronger than that of sessile polyps at the same low oxygen concentrations. As found in this experiment, the medusae decreased their frequency of bell contractions to compensate for the oxygen deficit at the first stage (0–30 hours) of hypoxic cultivation. During this period, the expression of the PHD-HIF oxygen-sensing system significantly changed, especially at 18 hours. The increasing levels of transcription factor HIF up-regulated the levels of ALDO to accelerate glycolysis or prevent the excess generation of ROS, up-regulated PDK to shut down oxidation, and up-regulated LDH to initiate anaerobic glycolysis. At the second stage (32~56 hours), the medusae temporarily adapted to the hypoxic environment by regulation of the PHD-HIF oxygen-sensing system, as shown mainly by the increase in the bell contraction rate and the high expression levels of *HIF*, *ALDO*, and *LDH*. At the third stage (58~72 hours), although the expression levels of *HIF*, *ALDO*, and *LDH* were increased, the bell contraction rate of the medusae significantly decreased again (Wilcoxon test, *p*-value < 0.01). The energy generated by glycolysis could not meet the physiological needs of the medusae in the absence of oxidative phosphorylation during this time, and high levels of lactic acid had negative effects on the physiological activity of the medusae. The above evidence suggested that the transition from aerobic respiration to anaerobic glycolysis, which is regulated by the PHD-HIF oxygen-sensing system, was the intrinsic mechanism governing the hypoxia response and tolerance in the medusae. For the polyps, the gene expression levels of *HIF*, *PHD*, and *ALDO* were slightly increased, while no obvious change was seen in *PDK* expression, and a small decrease in *LDH* expression was observed throughout the experiment. The observed up-regulation of *ALDO* suggested that glycolysis was increased. Predictably, although glycolysis was slightly improved, the levels of oxygen were sufficiently high for polyps to carry out oxidative phosphorylation in this experiment. As previously noted, the oxygen demand of pelagic medusae is greater than that of sessile polyps. Thus, the hypoxic tolerance of polyps is greater than that of medusae at the same low oxygen concentrations.

The decomposition of organic matter consumes DO in bottom waters, and stratification blocks the transport and mixing of oxygen between surface and bottom waters, resulting in oxygen depletion^36^. Oxygen depletion, called hypoxia, occurs in estuaries and coastal waters and represents the main threat to coastal ecosystems. The negative impacts of hypoxia include effects on marine organism populations, such as large-scale mortality and behavioural responses, as well as alterations in species distributions and biodiversity, increased physiological stress, and other sub-lethal effects. Our results verified that the polyps of *Aurelia* sp.1 can tolerate DO levels of 0.5 mg/L for 72 hours or even longer, as confirmed by Purcell’s studies on *Mnemiopsis leidyi* ^37^. In Tokyo Bay, substrates located in the hypoxic area are colonized by mussel populations in shallow waters and by polyps of *Aurelia* sp.1 in bottom waters^38^. Polyps have a higher survival rate in deeper, more oxygen-depleted waters than in surface waters with higher oxygen contents in the northern Gulf of Mexico^39^. Although DO levels of less than 0.5 mg/L have a detrimental effect on medusae, pelagic medusae can migrate vertically from the bottom to the surface or in the reverse direction in marine hypoxic areas^9, 40^, and the response period of 56 hours is sufficiently long to allow for multiple vertical migrations over the oxycline. This may represent a good presentation of the movement function during evolution. Furthermore, hypoxia can improve the predation rate of medusae as mentioned in the introduction; however, swimming speed and predation rate decrease in fish, such as Spanish mackerel (*Scomberomorus niphonius*) at oxygen concentrations <4.0 mg/L^4–6^. In summary, both polyps and medusae can tolerate marine hypoxia, which undoubtedly increases the competitive advantage of jellyfish in coastal ecosystems. Previous studies have shown that areas of prolonged marine hypoxia are consistent with areas where jellyfish gather, such as northern Hiroshima Bay, Tokyo Bay and the northern Gulf of Mexico^2, 38, 39^. Therefore, marine hypoxia may represent one of the key factors favouring jellyfish blooms worldwide.

## Conclusions

Under hypoxic conditions, physiological activity, the gene expression of PHD-HIF oxygen-sensing system-related genes and metabolism-related genes, and metabolite contents were significantly affected in medusae. When the incubation time was prolonged, the number of bell contraction first decreased (0~30 hours), then increased (32~56 hours), and finally decreased in a continuous manner (58~72 hours). The relative expression of *HIF* increased significantly from 3 hours to the highest value, which was observed at 18 hours and subsequently maintained. The variation in *PHD* and *ALDO* expression levels was similar to that in *HIF* expression levels during the experiment. After 6 hours, *PDK* expression increased to the highest value, which was observed at 18 hours, and gradually decreased thereafter. *LDH* expression also increased after 6 hours, and the lactic acid content increased after 18 hours. This evidence suggests that the transition from aerobic respiration to anaerobic glycolysis, which is regulated by the PHD-HIF oxygen-sensing system, regulates hypoxia response and tolerance in medusae. In polyps, there was a slight increase in the gene expression levels of *HIF*, *PHD*, and *ALDO*; however, *PDK* showed no clear change, and *LDH* decreased slightly throughout the experiment. This result suggests that 0.5 mg/L DO is sufficient for maintaining normal physiological function in polyps and that the transition from aerobic respiration to anaerobic glycolysis did not occur. This study provides a scientific basis for a thorough understanding of the regulatory mechanism of the PHD-HIF oxygen-sensing system in invertebrates and will help clarify the correlation between marine hypoxia and jellyfish blooms.

## Materials and Methods

### Experimental animal cultivation

*Aurelia* sp.1 (polyps and medusa) were provided by the Institute of Oceanography, Chinese Academy of Sciences (IOCAS). *Aurelia* sp.1 were sufficiently fed with *Artemia nauplius* every day and were maintained in 50-L tanks containing filtered seawater, which was replaced every day (salinity: 33, temperature: 19 °C, 12:12 light/dark cycle).

### Experimental design

The enclosed hypoxic jellyfish cultivation system consisted of a vitreous tank (A) and a loop system (B, C, D, E, and F) (Figure S1, supporting information). The vitreous tank was cylindrical (80 cm in diameter and 30 cm in thickness). The filtered seawater circulated along A, B, F, C, D, E and A. Two hypoxic cultivation systems were used in the hypoxic experiments. One was used as the reference system, in which DO was saturated through bubbling. The other system, in which approximately 0.5 mg/L DO was achieved by the bubbling of 99.9% nitrogen, was used for the hypoxic group. DO was measured every two hours using a Model HQ30d multi-parameter metre (HACH, Beijing, China) to maintain experimental stability. Thirty medusae of *Aurelia* sp.1 (umbrella diameter: ~4 cm) were used in the reference and hypoxic groups. Three medusae were randomly selected as samples in the reference and hypoxic groups at 0, 3, 6, 12, 18, 24, 36, 48 and 72 hours after the experiment onset. Each medusa in each sample was regarded as an experimental object. A polyethylene plate containing polyps was split into 16 pieces (4 × 4 cm), each of which was also included in the reference and hypoxic groups. One plate was randomly taken out for use as done in the medusa experiment, and 30 polyps per plate were randomly selected and equally divided into three parallel samples. Three biological replicates were examined in all experiments. We performed two hypoxic experiments: a medusa experiment and a polyp experiment. Each experiment was repeated more than two times independently. All other factors, including temperature, salinity, light regime, and feeding regime, were identical for both the experimental groups and non-treated controls to those factors before the experiment commenced.

### Physiological activity statistics

The number of medusa bell contractions was counted by direct observation every two hours during the experiment. At each time point, four medusae were randomly selected and the number of bell contractions was counted for 1 min.

### Total RNA extraction and reverse transcription

Total RNA was extracted from the medusa using Transzol (Transgen, Beijing, China), and the total RNA was extracted from polyps following the procedure described by Schroth *et al*.^41^. RNA quality was characterized by agarose gel electrophoresis and spectrophotometry. cDNA for use in quantitative PCR was obtained using the PrimeScript^TM^ RT reagent kit with gDNA Eraser (TaKaRa, Dalian, China).

### Real-time quantitative PCR experiments

The partial *PHD*, *ALDO*, *PDK*, and *LDH* nucleic acid sequences for *Aurelia* sp.1 were obtained from the RNA-seq (PRJNA219043) generated by IOCAS and were deposited into the nucleic acid sequence database at the National Center for Biotechnology Information (NCBI). Then, four pairs of quantitative primers (QALDO YF/YR, QPDK YF/YR, QPHD YF/YR and QLDH YF/YR) were designed based on their respective sequences using Primer 5.0 software. More information on the quantitative primers is listed in Table 1. The quantitative primer (Qtubulin YF/YR) for *HIF* was obtained from Wang *et al*.^16^. The internal control gene for RT-qPCR was *α-tubulin*, and this was verified to be a stable reference gene for the study of relative gene expression related to the hypoxic response of *Aurelia* sp.1 (unpublished data). The quantitative primers (Qtubulin YF/YR) used for *α-tubulin* are also listed in Table 1. Primers were synthesised by Biosune Biological Technology Co., Ltd. (Shanghai, China).

**Table 1:** List of quantitative primers used.

Primers name	Primer sequence (5′-3′)	Tm value (°C)	Amplified fragment length (bp)
Qtubulin YF	AGACAGAATCAGAAAGTTGGCAGA	63.2	220
Qtubulin YR	GTGAGTGGTCAGGATGGAGTTG	63.9	
QHIF YF	TATTTGATGGGCTGTTCTGCTC	62.0	280
QHIF YR	AGTAATGGGGTGCCAACTGCTA	65.1	
QALDO YF	ACCACAAACGAAACGACAACAC	63.6	219
QALDO YR	AGGCTCCACAATAGGCACCA	65.0	
QPDK YF	GCCTTGATAGCGGTAGTCCATA	62.7	200
QPDK YR	CGATTTGCCAAGAGTTGAAGTG	61.6	
QPHD YF	TGGTATTACGAGATTTGATGTGTTG	60.5	253
QPHD YR	TTCATCTTGCTTGCTGATACTTTGT	62.4	
QLDH YF	AGAAAACTGCTGCTGGATACAACT	63.8	289
QLDH YF	TGAAATTCAAGAAATAACGATGGAG	59.0	

The transcriptional profiles of the cDNA samples were quantified by PCR in a final reaction volume of 20 μL, including 10 μL of FastStart universal SYBR Green Master ROX (Roche, Mannheim, Germany), 2.0 μL of cDNA for quantitative PCR from *Aurelia* sp.1, 0.6 μL (10 μM) of quantitative primers, 2.0 μL (2 mg mL^−1^) of bovine serum albumin (TaKaRa, Dalian, China) and 4.8 μL of ddH_2_O. The templates were initially denatured at 95 °C for 10 min, followed by 40 cycles at 95 °C for 15 sec and 58 °C for 1 min, with a final extension at 72 °C for 10 min. The PCR was performed, and the amplification and dissociation curves of six primers were obtained using an ABI7500 fluorescence quantitative PCR instrument (Invitrogen, California, USA). The PCR products were directly sequenced using their own quantitative primers. For each primer pair, a dilution series (from 1/2 to 1/32) was created using a randomly selected cDNA sample. Furthermore, the logarithms of the cDNA dilution factors were plotted against the mean Ct values for each serial dilution to construct standard curves (Tables 2 and 3).

**Table 2:** Dilution curves of the tested medusa genes.

Genes	Standard curves(*R* ^*2*^)
*α-tubulin*	*y* = −3.22*x* + 25.23(0.999)
*HIF*	*y* = −3.28*x* + 31.17(0.996)
*ALDO*	*y* = −3.66*x* + 29.04(0.999)
*PDK*	*y* = −3.84*x* + 30.37(0.999)
*PHD*	*y* = −3.52*x* + 30.79(0.999)
*LDH*	*y* = −3.65*x* + 34.33(0.996)

**Table 3:** Dilution curves of the tested polyp genes.

Genes	Standard curves(*R* ^*2*^)
*α-tubulin*	*y* = −3.51*x* + 22.82(0.999)
*HIF*	*y* = −3.64*x* + 30.60(0.999)
*ALDO*	*y* = −3.64*x* + 26.63(0.999)
*PDK*	*y* = −3.90*x* + 29.71(0.999)
*PHD*	*y* = −3.72*x* + 31.81(0.998)
*LDH*	*y* = −3.72*x* + 31.89(0.998)

### Lactic acid detection

Lactic acid was detected using an LC-20A liquid chromatograph (Shimadzu, Japan). The chromatographic conditions used were as follows: mobile phase, 5% methanol in aqueous solution (containing 0.2% phosphoric acid, pH 2.5); flow velocity, 1.0 mL/min; injection volume, 20 μL; ultraviolet detection wavelength, 210 nm; column temperature, 30 °C; and ultraviolet detector, SPD-20A (Shimadzu, Japan). For details on the standard curve preparation, lactic acid recovery determination, and sample pretreatment, please see the supporting information.

### Statistical analyses

The Ct values were first converted into their respective dilutions based on the dilution standard curve for each gene. The ratio of target gene to reference gene quantity was then obtained for each sample. The relative expression (fold) of each target gene was then calculated based on the ratio of the quantity of an unknown sample versus that of the sample at 0 hours.

Statistical analysis and data presentation were performed using the R program (3.3.0). Differences between the control and hypoxia groups were identified using an independent sample t-test when the data were normally distributed; otherwise, the Wilcoxon rank sum test with continuity correction was applied. To study the regulatory mechanism, Pearson correlation coefficients were calculated for the ratio of the five different genes between the hypoxia and control groups for each sampling time in the medusae and polyps.

### Data availability

All EST sequences were submitted to National Centre for Biotechnology Information (NCBI) with accession numbers KX431206, KX431207, KX431208 and KX431209. Dissolved oxygen concentration, number of bell contractions, the raw qPCR data, and lactic acid content can be found in Dryad (http://dx.doi.org/10.5061/dryad.4gj62).

## Electronic supplementary material

Supporting information
