# Supplementary material for: The physiological and molecular response of Aurelia sp.1 under hypoxia
Source: Sci Rep. 2017 May 8;7:1558. doi: 10.1038/s41598-017-01318-x (PMC5431473; doi:10.1038/s41598-017-01318-x)
Supplement: Supplementary file 1 — Supporting information [file 41598_2017_1318_MOESM1_ESM.pdf]

The physiological and molecular response of *Aurelia* sp.1 under hypoxia

Guoshan Wang[1, 2, 3], Yu Zhen[1, 3, 4 #], Zhigang Yu [4, 5], Yan Shi[1], Qing Zhao[1,7], Jianyan Wang[6], Tiezhu Mi [1, 3, 4 *]

1. College of Environmental Science and Engineering, Ocean University of China, Qingdao, Shandong, P. R. China;

2. National Marine Hazard Mitigation Service, Beijing, China

3. Key Laboratory of Marine Environment and Ecology, Ministry of Education, P. R China;

4. Laboratory for Marine Ecology and Environmental Science, Qingdao National Laboratory for Marine Science and Technology, Qingdao, P. R. China;

5. Key Laboratory of Marine Chemistry Theory and Engineering, Ministry of Education, Shandong, P. R. China;

6. Beijing Museum of Natural History, Beijing, P. R. China;

7. Zhongtian Technology Marine Systems Co., Ltd., Nantong, P. R. China;

[#] Guoshan Wang and Yu Zhen contributed equally to this work.

Author for correspondence: Tiezhu Mi

E-mail: mitiezhu@ouc.edu.cn

238, Songling Road, Qingdao, Shandong Province, R. P. China

266100

Tel: +86 532 6678 1940

Fax: +86 532 6678 1940

Materials and methods

Standard curve preparation

A solution was prepared by adding 0.582 g of 86% lactic acid (Sigma-Aldrich, Beijing, China) to 100 mL of Milli-Q water; the final concentration of lactic acid solution was 5.01 g/L. The lactic acid solution was diluted to 200 mg/L, 100 mg/L, 50 mg/L, 20.0mg/L, 10.0 mg/L, 5.00 mg/L, and the serially diluted standards were analysed using chromatographic conditions described in the manuscript.

Determination of lactic acid recovery

After grinding under liquid nitrogen, a medusa sample (900 µL, fully mixed) was added to two 1.5-mL centrifuge tubes; then 100 µL of 500 mg/L lactic acid standard solution was added to one tube, and 100 µL of Milli-Q water was added to the other tube (each sample was analysed in triplicate). After vortexing, the samples were centrifuged at 4,000 × g for 5 minutes. Then, 900 µL of the supernatant was placed in a clean 1.5-mL centrifuge tube, and 100 µL of 10 M perchloric acid was added (Sinopharm Chemical Reagent Co. Ltd., Shanghai, China). The sample was centrifuged at 12,000 × g for 5 minutes after incubation at room temperature for 10 minutes; The supernatant was filtered through a 0.22-µm microfiltration membrane and analysed under the chromatographic conditions described in the manuscript.

Lactic acid detection of medusa samples

Medusa samples (1000 µL, fully mixed) were placed in 1.5-mL centrifuge tubes and centrifuged at 4,000 × g for 5 minutes. A total of 900 µL of the supernatant was then transferred to clean 1.5-mL centrifuge tubes, and 100 µL of 10 M perchloric acid was added. The reaction was mixed by swirling, and after incubation at room temperature for 10 minutes, the sample was centrifuged at 12,000 × g for 5 minutes. The supernatant was then filtered through a 0.22-µm microfiltration membrane and

analysed under the chromatographic conditions described in the manuscript.

Results

Lactic acid standard curve

The serially diluted standard sample concentrations (mg/L) and the peak areas in the chromatogram were subjected to linear fitting, yielding the following equation: $y=703.95x-299.16$ ($R^2=0.9999$) (Figure S2).

Lactic acid recovery

The average recovery rate of the jellyfish samples was 104.0±5.798% (Mean±SD) under the described chromatographic conditions; this rate was determined by measuring the lactic acid content before and after adding standard (Table S1). The results suggested that the described method was suitable for the detection of lactic acid in subsequent jellyfish samples.

Table

Table S1 The recovery results obtained in the lactic acid addition experiment

Sample	Lactic acid content before adding standard (mg/L)	The content of the added standard (mg/L)	Lactic acid content after adding the standard (mg/L)	Recovery rate (%)	Average recovery rate (%)
1	16.44	50.00	64.74	97.44	
2	17.07	50.00	71.29	106.3	104.0±5.798
3	14.27	50.00	69.64	108.4	

Figure captions

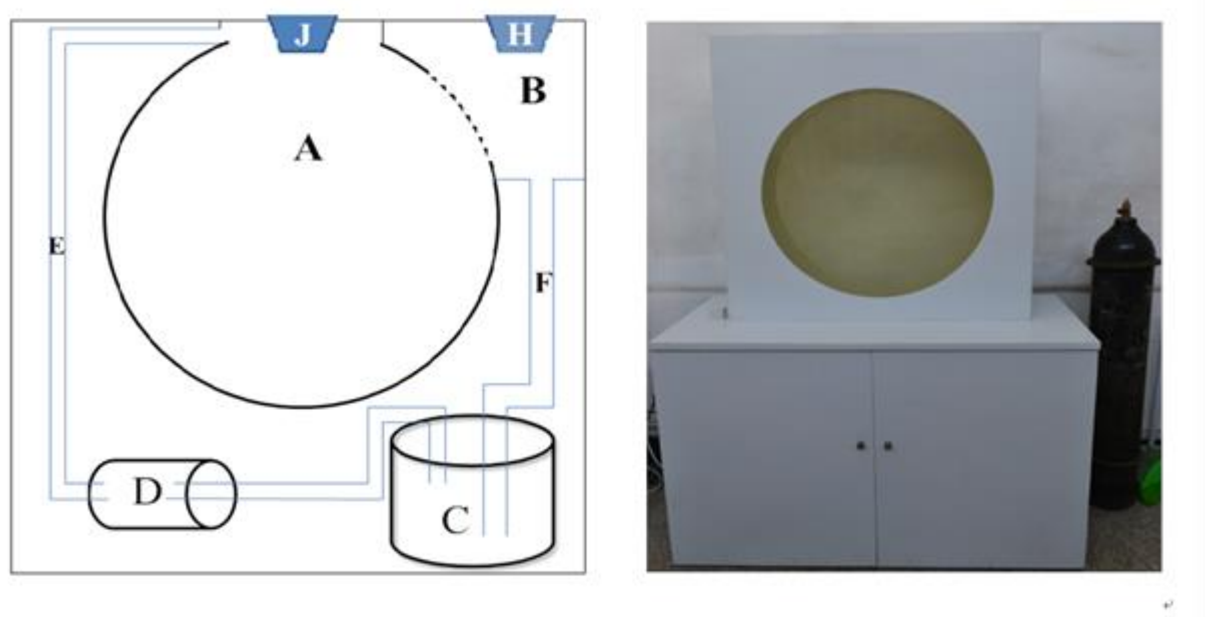

Figure S1 Structural diagram (left) and product shot (right) of hypoxic cultivation system (A-tank of jellyfish cultivation; B-buffer system; C-temperature controller; D-recycle pump; E-inlet pipe; F-outlet pipe; J&H-rubber plug).

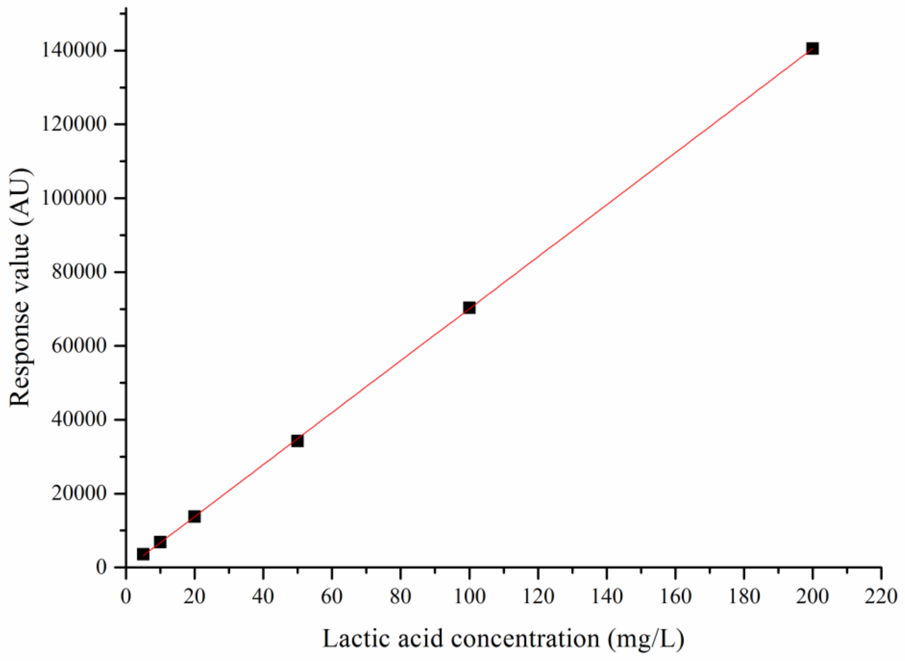

Figure S2 Standard curve for lactic acid detection
